# Single-cell transcriptome sequencing of plant leaf expressing anti-HER2 VHH–FcK cancer therapeutic protein

**DOI:** 10.1038/s41597-023-02833-5

**Published:** 2023-12-19

**Authors:** Myung-Shin Kim, Seung-Won Lee, Kibum Kim, Yerin Kim, Hyunjoo Hwang, Peter Hinterdorfer, Doil Choi, Kisung Ko

**Affiliations:** 1 Department of Agriculture, Forestry and Bioresources, Plant Genomics and Breeding Institute, College of Agriculture and Life Science, Seoul National University, Seoul, 08826 Korea; 2 Plant Immunity Research Center, Seoul National University, Seoul, 08826 Korea; 3 Department of Biosciences and Bioinformatics, Myongji University, Yongin, 17058 Korea; 4 Cancer Early Detection Advanced Research Center, Knight Cancer Institute, Oregon Health & Science University, Portland, Oregon 97201 USA; 5 Department of Medicine, College of Medicine, Chung-Ang University, Seoul, 06074 Korea; 6 Department of Applied Experimental Biophysics, Johannes Kepler University Linz, 4040 Linz, Austria

**Keywords:** Transgenic plants, Agroecology

## Abstract

The transgenic plant is a promising strategy for the production of highly valuable biotherapeutic proteins such as recombinant vaccines and antibodies. To achieve an efficient level of protein production, codon sequences and expression cassette elements need to be optimized. However, the systematical expression of recombinant proteins in plant biomass can generally be controlled for the production of therapeutic proteins after the generation of transgenic plants. Without understanding the transgene expression patterns in plant tissue, it is difficult to enhance further production levels. In this study, single-cell RNA-sequencing (scRNA-seq) analysis of transgenic tobacco (*Nicotiana tabacum*) leaf, expressing an immunotherapeutic llama antibody against breast cancer, anti-HER2 VHH–Fc, was conducted to obtain data on the expression pattern of tissue-specific cells. These high-quality scRNA-seq data enabled the identification of gene expression patterns by cell types, which can be applied to select the best cell types or tissues for the high production of these recombinant antibodies. These data provide a foundation to elucidate the mechanisms that regulate the biosynthesis of recombinant proteins in *N. tabacum*.

## Backgrounds & Summary

Breast cancer is globally the most diagnosed cancer in women. Herceptin is an immunotherapeutic monoclonal antibody (mAb) for breast cancer that overexpresses human epidermal growth factor receptor 2 (HER2)^1^. However, Herceptin is not always effective in breast cancer patients and could eventually cause Herceptin-resistant cancer^2^. HER2 is a tumor-associated antigen (TAA), which is targeted by antibodies to cause an anti-cancer immunotherapeutic effect. Thus, diverse anti-cancer immunotherapeutic antibody forms have been designed and expressed in heterologous systems^3^. Recently, llama heavy chain-only antibody that binds to HER2 (anti-HER2 VHH-Fc) has been produced to have several advantages such as small protein size and intrinsic stability for enhanced HER2 antigen binding activity^3,4^.

Plants are considered a heterologous bio-system for the production of valuable recombinant immunotherapeutic antibody proteins as an alternative to mammalian cell-based systems^5^. In plant expression system, the recombinant antibody genes are controlled by the CaMV35S promoter, which is considered a consistent and non-tissue-specific promoter in plant leaf and is mainly used for its stable or transient expression^6–8^. The transgene expression by the cauliflower mosaic virus (CaMV) 35S promoter has been investigated in leaf, root, and stem tissues^9^. Diverse CaMV35S promoter-controlled transgene transcription patterns were determined at leaf, root, and stem tissue levels^9^. However, specific profiles of the transgene expression under the control of the CaMV35S promoter have not been revealed at the single-cell level. It is essential to understand how tissue-specific cells perform transgene expression to improve the production of recombinant immunotherapeutic proteins in plant leaves. Recently, plant cell suspension biofactories have become popular in the production of biotherapeutic proteins^10^. Thus, the application of well-characterized leaf cell types and conditions for high transgene expression is essential for developing a cell suspension factory system that can efficiently produce recombinant proteins^11^. However, unlike unicellular organisms, each plant tissue consists of a different cell type, whereby the leaf is composed of mainly mesophyll, vasculature, and epidermis cells^12^. It is important to determine the expression of the CaMV35S promoter-controlled transgene in each leaf type to understand whether the transgene has concordant expression levels among the different cell types. This central question in transgenic expression patterns at the plant leaf single-cell level has yet to be answered, which is essential to determine how to enhance the production of valuable recombinant proteins in plant molecular biopharming system.

Single-cell RNA sequencing (scRNA-seq) technology has been developed to provide unlimited opportunities to reveal gene expression profiles at single-cell levels^13^. For successful scRNA-seq-analysis, it is essential to generate abundant viable protoplast from cell suspensions. However, the differentiation of plant cell walls by variable factors, including plant tissues, and developmental stages, even in a single plant biomass, can hamper obtaining cell-type-specific unbiased protoplasts suitable for scRNA-seq^14^. In general, an insufficient number of viable, high-quality protoplasts have been applied to scRNA-seq resulting in biased and unreliable data interpretations^15^.

**Fig. 1:**
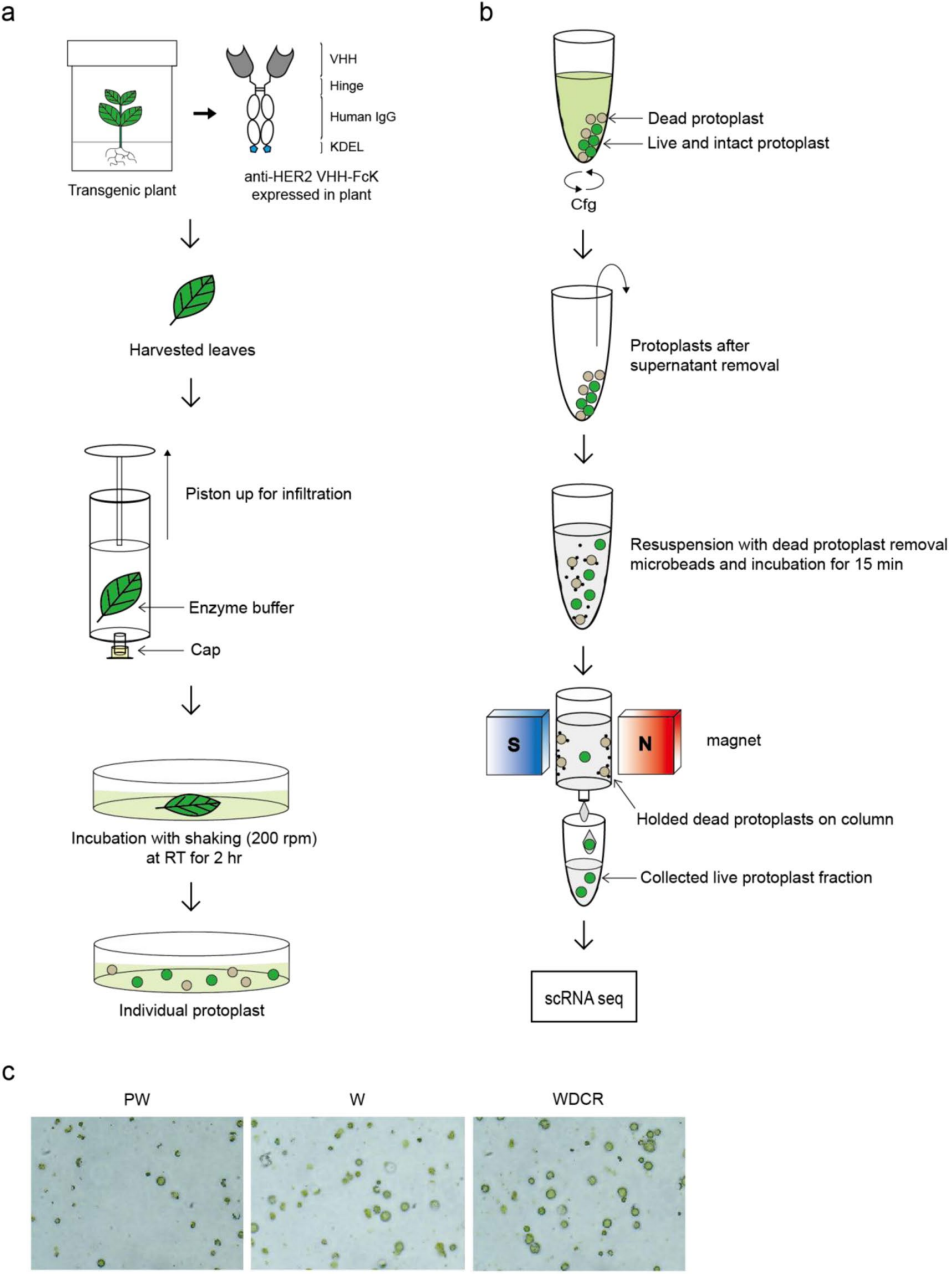
Workflow of highly efficient leaf protoplast isolation for scRNA-seq analysis. (**a**) Leaf protoplasts for scRNA-seq analysis were isolated from *in vitro* transgenic tobacco plant leaves using a protoplast isolation buffer containing plant cell wall lytic enzymes and a dead cell remover (DCR) kit. The harvested leaves (200~300 mg) were infiltrated with enzymes using a 50 mL syringe and incubated in a Petri dish at room temperature for 2 hrs. (**b**) The dead protoplasts and debris were labeled with microbeads and retained in the magnetic field, whereas the live protoplasts were rinsed out and collected for the scRNA-seq analysis. (**c**) The collected protoplasts obtained from different isolation protocols. PW, pre-washing group, where the washing step was omitted; W, washing group, where the washing step was included; WDCR, dead cell remover group, where the washing and dead cell removing steps were included. Microscopic analysis was conducted to count the viable protoplast number in the sample obtained using the highly efficient leaf protoplast isolation protocol (WDCR).

In this study, the protocol for robust protoplast isolation from tobacco (*Nicotiana tabacum*) expressing anti-HER2 VHH-FcK was optimized for scRNA-seq analysis. In our previous study, we generated a transgenic tobacco plant, which expressed anti-HER2 VHH–FcK fusion proteins under the control of a CaMV35S promoter^3^ (Fig. 1a). The transgenic tobacco leaf tissues were confirmed to have the mRNA and protein expression of anti-HER2 VHH–FcK gene. The viable protoplasts were isolated and separated from transgenic tobacco leaf using enzyme buffer and a magnetic field, respectively, and were counted under a microscope for single-cell transcriptome analysis (Fig. 1). In this study, a method that isolated only live protoplasts from a mixed sample solution of dead and live protoplasts was optimized and established in plant system (Table 1 & Fig. 1). Furthermore, the live protoplasts were applied to scRNA-seq analysis (Table 1, Fig. 1, & Fig. S1). The method of yielding high numbers of plant protoplasts that could survive from a leaf was followed by an additional isolation step, which used a dead cell removal (DCR) kit (Fig. 1b). The addition of this step both removes unwanted components, such as the dead protoplasts and cell debris and allows for the acquisition of high-yield viable plant protoplasts, maintaining their intact form and function. Therefore, this method could be effectively utilized in various applications that use plant cells and require high cell viability and plant transformation experiments, such as single-cell analysis.

**Table 1:** Protoplast viability with different leaf protoplast processing protocols.

Process^z^	Total protoplast conc (cells/mL)	Live protoplast conc (cells/mL)	Viability (%)^y^	Final amount of live protoplast^x^
PW	1.6 × 10^6^	7.8 × 10^5^	49%	7.8 × 10^5^
W	2.8 × 10^5^	2.2 × 10^5^	78%	2.2 × 10^5^
WDCR	3.7 × 10^5^	3.4 × 10^5^	92%	0.6 × 10^5^

^z^PW (Pre-washing): Protoplasts isolated from only one step of enzyme infiltration. W (Washing): Protoplasts isolated from two steps of enzyme infiltration and enzyme removal by washing. WDCR (Washing with DCR): Protoplasts isolated by washing and DCR (dead cell remover) steps.

^y^Viability (%) was calculated using a formula: 100 × [live protoplast concentration (cell/mL)/total protoplast concentration (cell/mL)].

^x^Final amount of live protoplast: The final number of live protoplasts obtained from each process group.

**Fig. 2:**
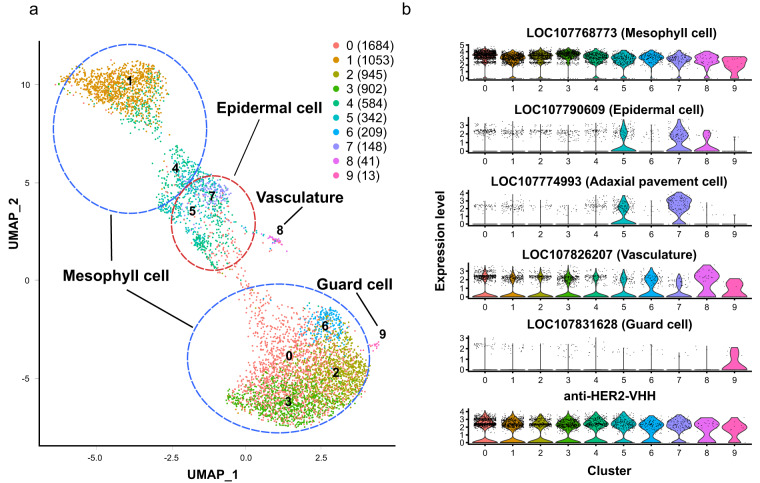
Gene expression landscape of leaf cell populations in transgenic tobacco (*Nicotiana tabacum*) using scRNA-seq. (**a**) Uniform manifold approximation and projection (UMAP) plot showing the four main cell types of leaf cell populations. (**b**) Violin plot of selected marker genes and anti-HER2 VHH-FcK gene. Each marker gene represents, from top to bottom, mesophyll, epidermal, adaxial pavement, vasculature, and guard cell types and the anti-HER2 VHH-FcK gene, respectively.

Using the 10x genomics scRNA-seq platform, we generated scRNA-seq data containing 7,740 leaf cells (Table 2). After quality control, 5,921 high-quality cells with 20,358 expressed genes were grouped into 9 clusters (Fig. 2a). These clusters were assigned cell types through homology-based marker gene predictions by PlantscRNAdb^16^ and previously reported Arabidopsis marker genes^17,18^ (Fig. 2, Fig. S2 & Table S1). In the expression of the anti-HER2 VHH–FcK gene, all the cell-type clusters were evenly expressed, which suggests that all cell types are useful for biofactory (Fig. 2b). These data offer precise insights into promoter functions within leaf tissues, particularly in specific cell types. Additionally, they provide guidance on identifying leaf tissues with high levels of recombinant immunotherapeutic antibodies. Furthermore, the identified cells with the highest transgene expressions can be isolated and utilized in the cell suspension factory system to produce valuable recombinant proteins.

**Table 2:** Statistics of scRNA-seq dataset used in this study.

	HER2
Number of reads	402,787,243
Estimated number of cells	7,740
Median number of genes per cell	1,088
Median number of UMI counts per cell	1,665
Mean reads per cell	52,040
Q30 bases in barcode	97.30%
Q30 bases in RNA read	91.90%
Q30 bases in UMI	97.30%
Reads mapped to genome	93.00%
Reads mapped confidently to genome	58.00%
Reads mapped confidently to exonic regions	50.70%
Reads mapped confidently to transcriptome	49.30%

## Materials and Methods

### Plant leaf sample collection

The anti-HER2 VHH–FcK gene expression was controlled by the CaMV35S promoter with which contained duplicated upstream B domains (Ca2 promoter) and the untranslated leader sequence of alfalfa mosaic virus RNA4 in the pBI121 plant binary vector. Transgenic tobacco plants expressing a llama single-domain anti-HER2 antibody with KDEL (anti-HER2 VHH–FcK) were generated by *Agrobacterium*-mediated transformation and *in vitro* tissue-cultured in MS medium with kanamycin (100 mg/L). Transgenic tobacco plants were grown in a plant growth chamber under optimized conditions (130 μmol photons m^−2^ s^−1^ at 23 °C with a relative humidity of 70% and a 16/8 h light/dark cycle). Likewise, the non-transgenic tobacco plants were also grown in the same plant growth chamber.

### Protoplast isolation, library construction, and scRNA-sequencing

Leaf protoplasts were isolated from *in vitro* transgenic tobacco plants (Fig. 1). Leaves were immersed in protoplast isolation buffer [3 mM MES, 7 mM CaCl_2_, 0.4 M mannitol, and KOH are used for adjusting buffer pH 5.8] and enzyme buffer [1.5% (w/v) cellulose Onozuka R-10 and 0.5% (w/v) macerozyme in protoplast isolation buffer]. Experiments were performed by harvesting 4–5 cm tobacco plant leaves cultured in sterile conditions. The harvested leaves were placed in a 50 mL syringe with 20 mL enzyme buffer, and the enzyme was infiltrated into the leaves by vacuum evacuation (Fig. 1a). The leaves infiltrated with enzyme buffer were shaken at 200 rpm for 2 hours at room temperature with 5 mL enzyme buffer and filtered through 70 μm and 40 μm filters. The filtered protoplasts were centrifuged at 500 rpm for 10 minutes and washed with protoplast isolation buffer. The dead protoplasts and debris, labeled with microbeads, were retained in the column using a magnetic field, while the live protoplasts were released from the columns (Fig. 1b) using the dead cell removal kit (Miltenyi Biotec, Seoul, Korea). The cell concentration and viability were calculated using a Cellometer Auto 2000 (Nexcelom Bioscience, USA) with 0.2% trypan blue. Subsequently, the buffer was exchanged with the loading buffer and approximately 20,000 transgenic protoplasts were loaded into a 10x Chromium Controller (10x Genomics, Pleasanton, CA) to generate single-cell GEMs (gel beads in the emulsion). The library was constructed using Chromium Next GEM Single Cell 3’ Kit v3.1 (10x Genomics). Sequencing was performed by Illumina HiSeq 4000, according to the manufacturer’s instructions (Illumina).

### Tissue extraction, library construction, and bulk RNA-sequencing

Leaf tissues were harvested from *in vitro* transgenic plants, ground in liquid nitrogen, and applied to isolate total RNA using TRI Reagent™ solution^19^. One μg of mRNA was applied to construct the cDNA libraries using TruSeq Stranded mRNA Library Prep Kit (Illumina). The generated cDNA libraries were quantified using the qPCR Quantification Protocol Guide (Illumina). The template size distribution (300~500 bp) was confirmed to verify the PCR-enriched fragment sizes using a 2100 Bioanalyzer (Agilent Technologies). The library sequencing was conducted using the Illumina NovaSeq 6000.

### Generation of single-cell and bulk RNA-seq datasets

For both scRNA-seq and bulk RNA-seq data analyses, the reference genome and gene annotation of *N. tabacum* TN90 were downloaded from the RefSeq database (GCF_000715135.1)^20^. The anti-HER2 VHH gene sequence was obtained from Dr. Brigitte Kerfelec, French National Institute of Health and Medical Research. To identify homologous genes between *N. tabacum* and *Arabidopsis thaliana*, whole protein sequences of *N. tabacum* TN90 and *A. thaliana* Araport11 (version Jan192022)^21^ were clustered using OrthoFinder v.2.5.4 (-S blast)^22^. Using the scRNA-seq data, we performed the 10x Genomics Cell Ranger v.6.0.2 pipeline with default parameters^23^. Sequencing reads were mapped to the reference genome with the anti-HER2 VHH–Fc gene sequence and a feature-barcode matrix was generated using the count command implemented in Cell Ranger. Using the bulk RNA-seq data, sequencing reads were filtered in Trimmomatic v.0.39^24^ with ILLUMINACLIP:TruSeq 3-PE.fa:2:30:10 TRAILING:3 SLIDINGWINDOW:4:15 MINLEN:36 parameters. The trimmed reads were aligned to the reference genome using HISAT v.2.2.1^25^ with default parameters. The mapped reads were quantified and counted using Stringtie v.2.2.1^26^ with default parameters and prepDE.py3 implemented in Stringtie with –l 100 parameters, respectively.

### Quality control of scRNA-seq data

The filtered feature-barcode matrix was imported into Seurat v.4.1.1^27^ for all downstream analyses. Quality control was performed to filter out any low-quality cells and genes. Only protein-coding genes were kept to filter out the uninformative pseudogenes and non-coding RNAs. To eliminate potential doublets, we visualized a boxplot of the number of expressed genes per cell (nGene), and then, calculated the upper whisker, which is the maximum value excluding outliers, using the boxplot()$stats function in R software v.4.1.3 (https://www.r-project.org/). Since the value of the upper whisker was 2,800, only cells with a nGene score of more than 300 and less than 3,000 were retained. We also removed any cells with 1) a mitochondrial gene ratio of more than 5%; 2) a chloroplast gene ratio of more than 25%; 3) a log_10_-transformed ratio of the nGene and a number 0.9 or less for the unique molecular identifiers (nUMI). To filter out stress-related genes induced by protoplast isolation, we compared the gene expressions between scRNA-seq and bulk RNA-seq datasets, similar to previously reported methods^28^. Pseudo-bulk data were generated using a row-summed scRNA-seq counts matrix. Pearson’s correlation coefficient of gene expression between the pseudo-bulk scRNA-seq and bulk RNA-seq datasets was calculated. Genes with a log_2_ fold change greater than 5 in scRNA-seq data than in bulk RNA-seq data were considered stress-related genes and removed. Genes less than 50 UMIs in the pseudo-bulk scRNA-seq data were defined as lowly expressed genes and also removed.

### Clustering and gene expression profiling of scRNA-seq data

The gene expression of filtered scRNA-seq data was log-normalized by NormalizeData implemented in Seurat^27^. The cell cycle score for each cell was calculated by CellCycleScoring implemented in Seurat^27^. Cell cycle gene homologs were identified by protein clusters generated from OrthoFinder v.2.5.4^22^ and known cell cycle genes in *A. thaliana*^29,30^. The normalized scRNA-seq data were transformed using SCTransform (vst.flavor = “v2”)^31^. Mitochondrial and chloroplast gene ratios per cell and cell cycle phase were regressed during the transformation step. Principal component analysis (PCA) was performed using RunPCA implemented in Seurat^27^ to calculate 100 principal components (PCs). All 100 PCs were used for uniform manifold approximation and projection (UMAP) embedding by RunUMAP implemented in Seurat^27^. Additional potential doublets were identified and removed using DoubletFinder v.2.0.3 with default parameters^32^. Clustering was conducted using FindNeighbors (reduction = “pca”, dims = 1:100) and FindClusters (resolution = 1.0). The gene expression patterns of nUMI, nGene, mitochondrial, and chloroplast gene ratios per cell were visualized using FeaturePlot (reduction = “umap”, min.cutoff = “q10”).

To assign putative cell types in each cluster, differentially expressed genes (DEGs) per cluster were identified using FindAllMarkers (only.pos = TRUE, min.pct = 0.30, logfc.threshold = 0.25). Known marker genes of leaf in *A. thaliana* were collected from PlantscRNAdb^16^ and previous studies^17,18^. Homology-based marker genes between *N. tabacum* and *A. thaliana* were identified from protein clusters generated by OrthoFinder v.2.5.4^22^. The homology-based marker genes and the DEGs were combined, and the cell type was assigned for each cluster. Representative marker genes were visualized using VlnPlot implemented in Seurat^27^.

## Data Records

The scRNA-seq and bulk RNA-seq data used in this study are available on NCBI under the BioProject accession PRJNA970858. The sample descriptions and raw sequencing data for scRNA-seq and bulk RNA-seq are available under the BioSample accession numbers (SAMN35016658 and SAMN35016659, respectively), and the Sequence Read Archive (SRA) accession number SRP436736 (SRR24488786 and SRR24488787, respectively)^33^. The final gene expression profiling and cluster numbers for each cell had been deposited in FigShare^34^.

## Technical Validation

### Protoplast viability

After obtaining the plant cells through the methods described above, their survival rates were analyzed. Three groups were classified: the pre-washing (PW) group, where the washing step from the conventional plant protoplast extraction method was omitted; the washing (W) group, where the washing step from the conventional method was applied; the washing with DCR (WDCR) group, where the conventional method was applied, followed by washing step and the isolation of the live protoplasts using the DCR kit. To calculate the viability of protoplasts, the total protoplast concentration, viable protoplast concentration, and resuspension volume were measured for each group, and the viability was determined using the following formula: Viability (%) = 100 x [viable protoplast concentration (cells/mL)/total protoplast concentration (cells/mL)]. The results showed that the WDCR group had the highest viable protoplast rate, which was followed by the W and PW groups (Table 1). Moreover, the PW group had the lowest total viable protoplast concentration. Furthermore, the W group had the highest total protoplast concentration but a moderate concentration of viable protoplasts. Finally, although the total protoplast concentration was low in the WDCR group, it exhibited the highest viable protoplast concentration. Comparing the viability values, the PW group had a viability of 49%, the W group had a viability of 78%, and the WDCR group had a viability of 92%, which was approximately 2.2 and 1.2 times higher than the PW and W groups, respectively. Particularly, despite the slightly lower final protoplast amount, the viable protoplast concentration was the highest when using the DCR kit, which is ideal for scRNA-seq analysis (Table 1). A resuspension buffer solution was used to alleviate the re-aggregation of protoplasts after protoplast extraction. In this case, the volume of resuspension buffer used in the DCR group was approximately 1/25 and 1/5 compared to the PW and W groups, respectively. This indicates that the viability of protoplasts increased without the need for large amounts of reagents to address the re-aggregation of protoplasts after extraction. The method includes an additional isolation step, where the dead cell removal kit was used after using conventional methods to extract the protoplasts from the plant. This protoplast isolation method was ideally optimized to increase viability of protoplasts for scRNA-seq analysis.

### Quality assessment of sequencing data

We generated scRNA-seq and bulk RNA-seq datasets for anti-HER2 VHH-FcK transgenic and wild type tobacco leaves, respectively. Approximately 402 million read pairs were obtained in the scRNA-seq data, of which, a proportion of 93.19% had a quality score of 30 (Table 2 & Table S2). These sequencing read pairs were mapped to the *N. tabacum* genome with a 93% mapping ratio. At the single-cell resolution, a total of 7,740 cells were obtained with a median number of 1,088 genes and an average of 52,040 reads per cell (Table 2). In the bulk RNA-seq data, about 37 million read pairs were sequenced, with a proportion of 94.44% having a higher quality score of 30 (Table S2). After quality trimming, 36 million read pairs were retained and mapped to the *N. tabacum* genome with a 97% mapping ratio (Table S2). Overall, these results indicate that high-quality sequencing data were generated in both the scRNA-seq and bulk RNA-seq datasets.

**Fig. 3:**
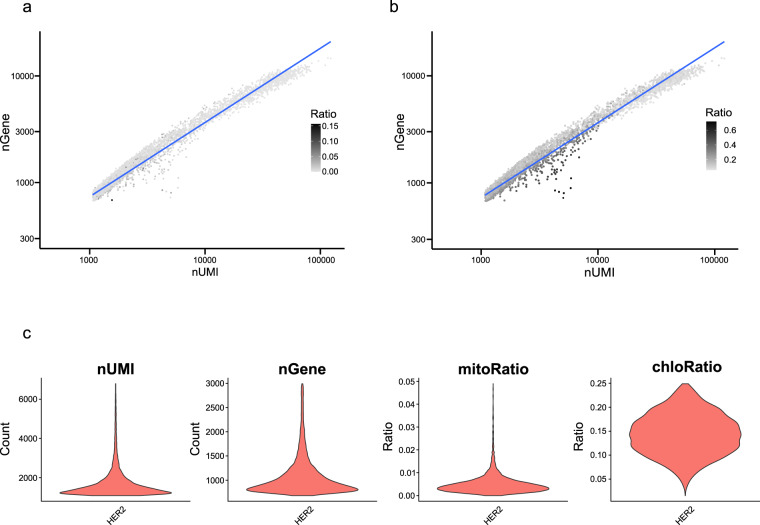
Quality control of scRNA-seq data in transgenic tobacco leaves. Scatter plot between the number of unique molecular identifiers (nUMI) and genes (nGene) with (**a**) expressed mitochondrial gene ratio and (**b**) chloroplast gene ratio per cell. The blue line represents the correlation ratio between nUMI and nGene. (**c**) Violin plots representing the nUMI, nGene, and the percentage of mitochondrial and chloroplast genes in filtered scRNA-seq data.

To optimize the quality control cutoff, we visualized the nGene density, the mitochondrial and chloroplast gene expression proportions, and the complexity defined by the ratio between nGene and nUMI per cell in the scRNA-seq data. Consequently, we identified potential doublet cells and low complexity cells that contained high proportions of mitochondrial and chloroplast genes (Fig. 3a,b, and Fig. S3a–c). In addition, we generated a pseudo-bulk scRNA-seq dataset that combined the sum of each gene expressed in each cell of scRNA-seq and compared the correlation between pseudo-bulk scRNA-seq and bulk RNA-seq datasets to eliminate potential stress-responsive genes caused by protoplast isolation and transgene; ultimately, finding that the expression patterns between them were highly correlated (Pearson’s correlation, *r* = 0.72; Fig. S3d). A total of 1,838 genes with a log_2_ fold change greater than 5 in pseudo-bulk scRNA-seq over bulk RNA-seq were removed, including ethylene-responsive transcription factors and zinc-finger proteins induced by protoplast isolation^28,35^ (Table S3). Using these stress-responsive genes, we filtered out low-quality cells, genes, and potential doublets, which left 5,921 cells, with 20,358 expressed genes, and confirmed the expression patterns of the mitochondrial and chloroplast genes, nUMI, and nGene in the filtered scRNA-seq data (Fig. 3c). The median values for nUMI and nGene, in the mitochondrial and chloroplast gene percentages, were 1,172 and 859, respectively, and 0.004 and 0.143, respectively. These results suggest that high-quality scRNA-seq data were obtained to identify the cell-type specificity of leaves from *N. tabacum*.

### Gene expression profile and cluster identification of scRNA-seq data

**Fig. 4:**
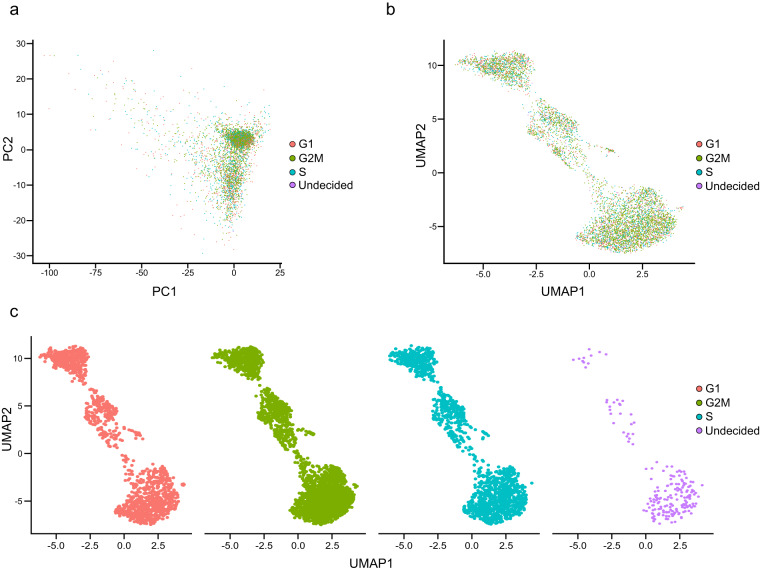
Cell cycle phase distribution of transgenic tobacco leaves. (**a**) Principal component analysis (PCA) and (**b**) and (**c**) UMAP plots are colored by the G1, G2/M, and S-phases of the cell cycle. Cells with an unclear cell cycle score are indicated as “Undecided”.

**Fig. 5:**
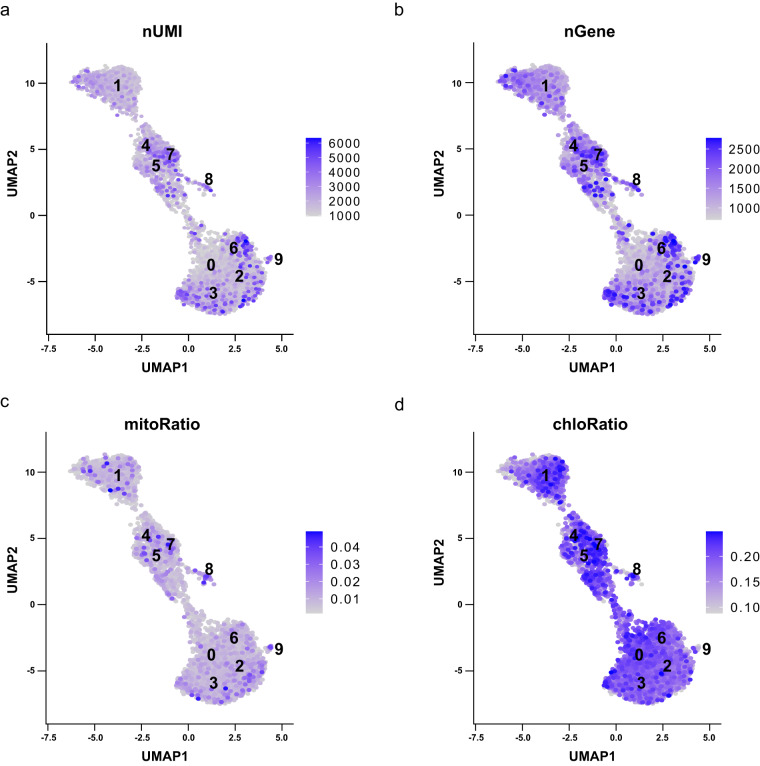
Quality evaluation of clusters for four features. Each feature plot represents (**a**) nUMI, (**b**) nGene, (**c**) mitochondrial gene ratio per cell, and (**d**) chloroplast gene ratio per cell.

To profile the gene expression patterns and assign cell types in a leaf cell population, filtered scRNA-seq data were normalized and clustered. Before clustering, cell cycle scoring was performed to reduce biased clustering caused by the cell phase. We identified 28 and 10 cell cycle gene homologs for the G1/S and G2/M-phases, respectively, using known cell cycle genes in *A. thaliana*^29,30^ (Table S4). Cell cycle scoring, PCA, and UMAP analyses revealed that cells were evenly distributed without separation by cell cycle phase, reducing the effects of cell-cycle heterogeneity (Fig. 4). Subsequent transformation and clustering processes grouped the cells into nine clusters (Fig. 2a). To confirm the global gene expression pattern, we visualized the gene expressions of four features (nUMI, nGene, and mitochondrial and chloroplast ratios per cell). The two features of nUMI and nGene were differently expressed among clusters, while the other two features (mitochondrial and chloroplast ratio per cell) were expressed relatively evenly without a specific cluster containing cells with a high number of expressed genes (Fig. 5). These results indicate that potential doublets are successfully removed, and these clusters are sufficient to identify cell-type specificity.

### Annotation of cell-type specific clusters

We assigned cell types to nine clusters using homology-based markers from *A. thaliana* (Table S1). Of the nine clusters, six clusters with 5,377 cells and were assigned to mesophyll cells, and the remaining clusters with 490, 41, and 13 cells were assigned to epidermal, vasculature, and guard cells, respectively (Fig. 2a). LOC107768773 and LOC107831628 genes, which are homologs of CARBONIC ANHYDRASE 1 (CA1, AT3G01500)^17^ and SCREAM (SCRM, AT3G26744)^18^ in Arabidopsis, were specifically expressed in the mesophyll and guard cell clusters, respectively. Other cell type clusters also expressed marker gene homologs derived from PlantscRNAdb (Fig. 2b & Fig. S2). These results indicate that our scRNA-seq data successfully identified clusters by cell type. Successful mapping of a single-cell transcriptomic landscape in plant leaf tissue depends on the dissociation of the tissue into live and intact single cells and equal cell separation without spatial and morphological bias. In this study, leaf protoplasts were successfully isolated from *in vitro* transgenic tobacco plants to provide crucial insight into the location of specific cells expressing transgenes and established a robust cell isolation protocol for scRNA-seq analysis.

### Supplementary information

Supplementary Information

Table S1

Table S2

Table S3

Table S4

## Data Availability

The script used in this study was deposited in GitHub (https://github.com/Myung-ShinKim/scRNA-seq_pipeline).
