# Supplementary material for: Single-cell transcriptome sequencing of plant leaf expressing anti-HER2 VHH–FcK cancer therapeutic protein
Source: Sci Data. 2023 Dec 19;10:911. doi: 10.1038/s41597-023-02833-5 (PMC10730532; doi:10.1038/s41597-023-02833-5)
Supplement: Supplementary file 1 — Supplementary Information [file 41597_2023_2833_MOESM1_ESM.pdf]

Supplementary Information

Table S1 (separated file)
Table S2 (separated file)
Table S3 (separated file)
Table S4 (separated file)

Figure S1
Figure S2
Figure S3

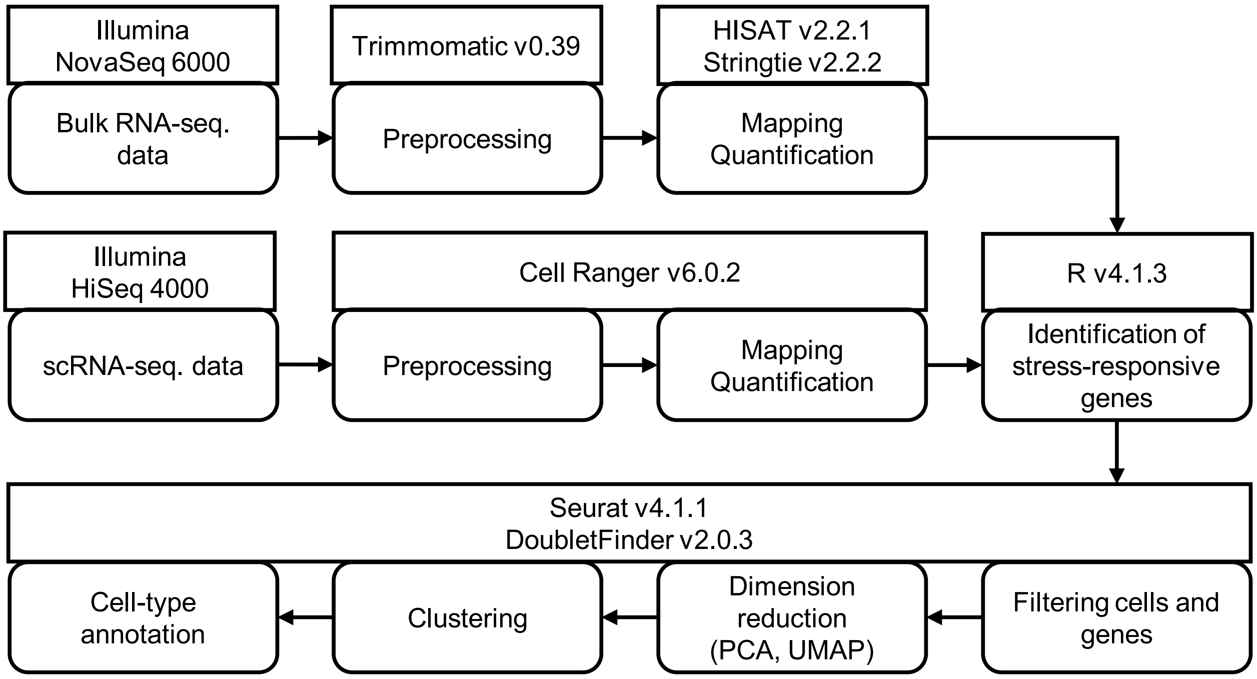

Figure S1. Overview of the scRNA-seq analysis pipeline.

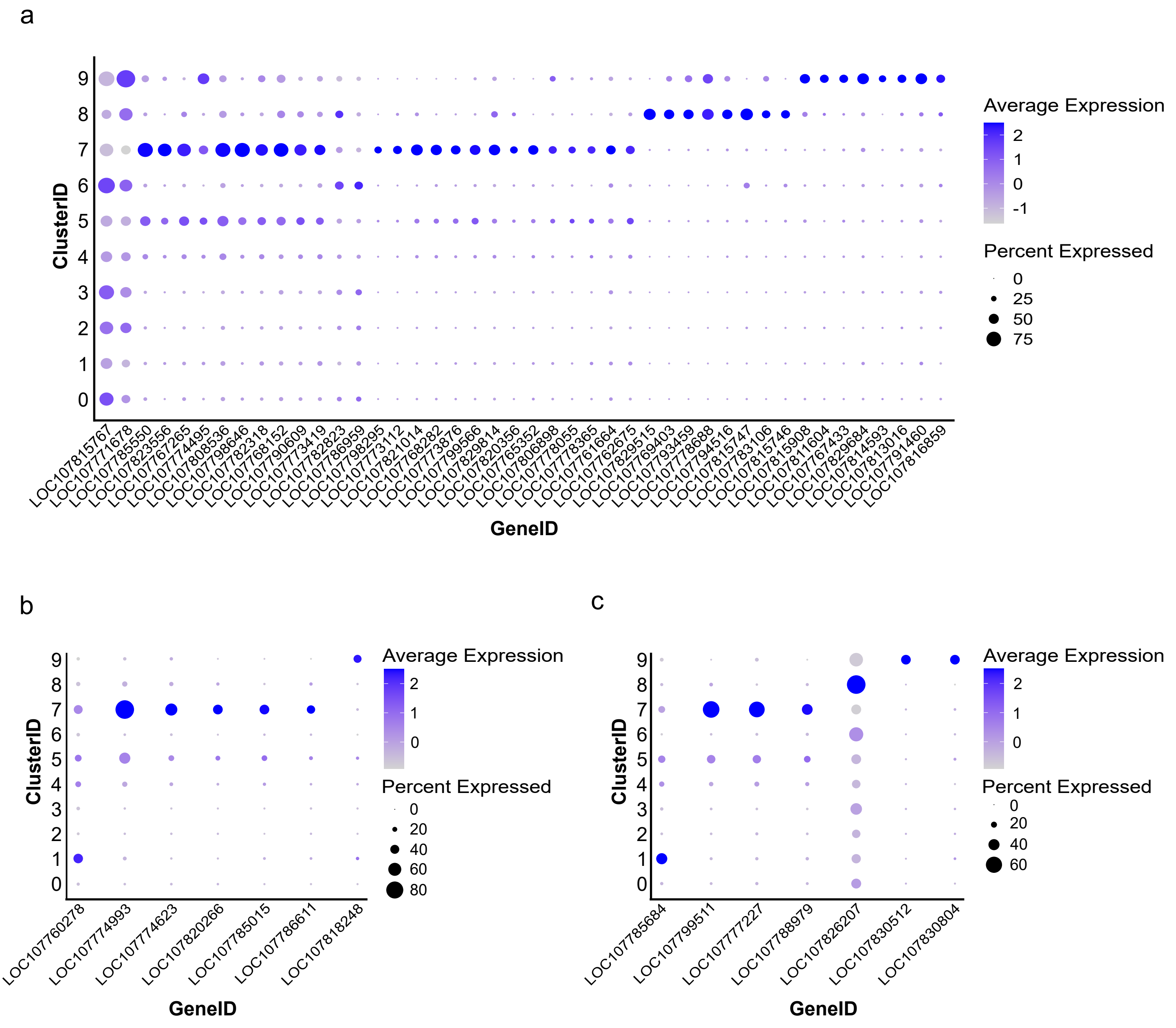

Figure S2. Dot plots of marker genes by cluster using PlantscRNAdb marker genes. The a) epidermal, b) adaxial pavement, and c) vasculature cell types are depicted.

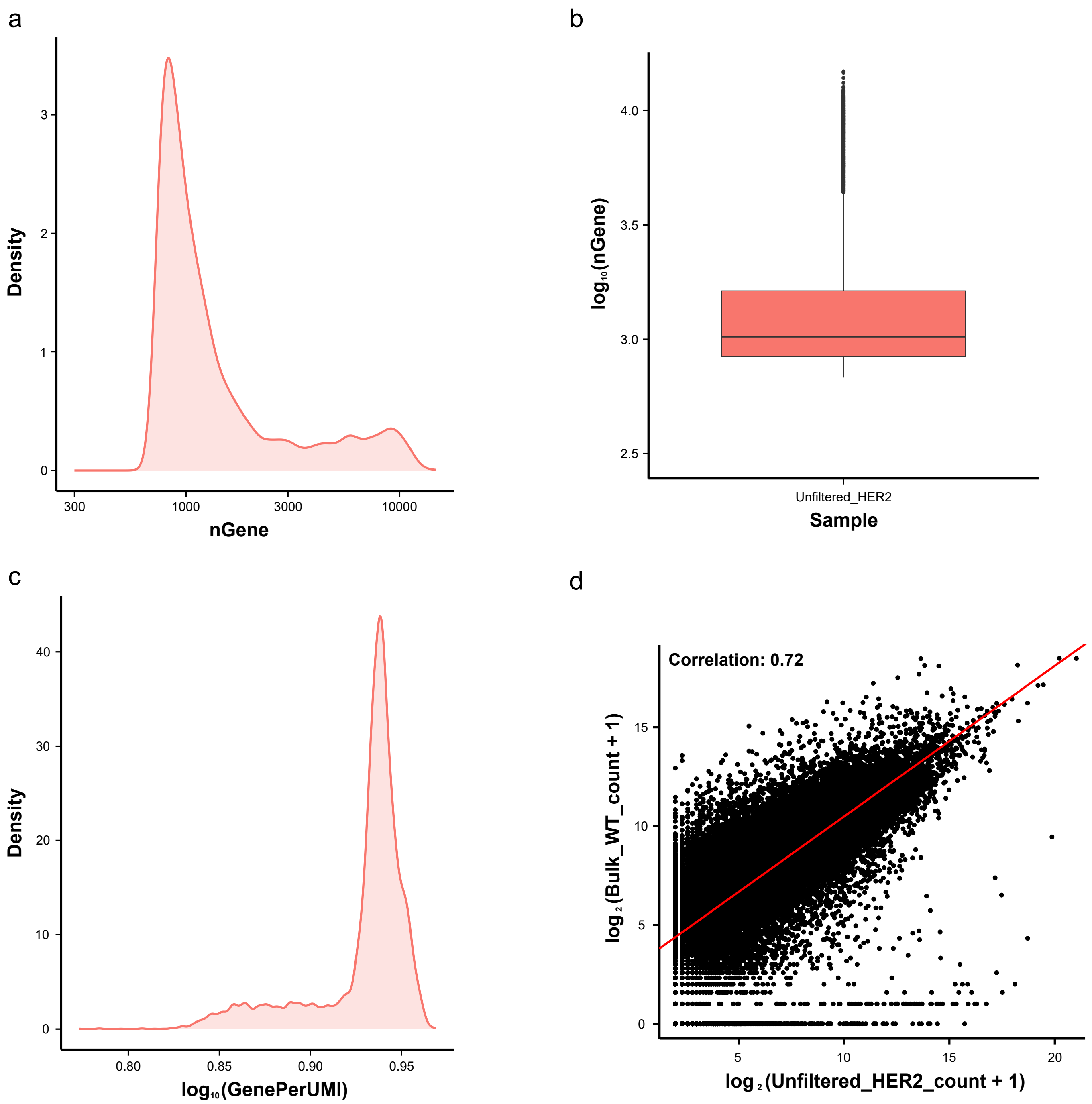

Figure S3. Expression profiles in the unfiltered HER2 dataset. a) density and b) box plots illustrating the number of expressed genes (nGene). c) Density plot of gene per UMI ratio. d) Scatter plot of gene expression between pseudo-bulk scRNA-seq and bulk RNA-seq datasets. The red line represents Pearson’s correlation coefficient between the two datasets.
